# Phylogeny of the subgenus *Eumitria* in Tanzania

**DOI:** 10.1080/21501203.2019.1635217

**Published:** 2019-06-30

**Authors:** Stella G. Temu, Philippe Clerc, Leif Tibell, Donatha D. Tibuhwa, Sanja Tibell

**Affiliations:** a Department of Organismal Biology, Uppsala University (UU), Uppsala, Sweden; b Dept. of Molecular Biology and Biotechnology, University of Dar es Salaam (UDSM),Tanzania; c Conservatoire et Jardin Botaniques de la Ville de Genève (CJBG), Geneva, Switzerland

**Keywords:** Lichens, molecular phylogeny, morphology, secondary chemistry

## Abstract

Several *Usnea* species in subgenus *Eumitria* (*Parmeliaceae*, lichenized Ascomycota) have been described from East Africa in the past decades. These have been based on morphology and chemistry data while molecular studies remain very limited. In this paper we are for the first time publishing phylogenetic analyses along with morphological and chemical data for *Eumitria*. ‬A total of 62 new sequences of *Eumitria* (26 ITS, 20 nuLSU, 6 MCM7, 10 RPB1) were generated in this study. nuLSU, MCM7 and RPB1 sequences are here for the first time reported for *U. baileyi*. A phylogeny of subgenus *Eumitria* from Tanzania based on Bayesian and maximum likelihood analyses of a concatenated four-loci data set is presented, confirming the monophyly of *Eumitria*. Further, secondary chemistry and variation in characters, such as the pigmentation of the central axis and branch shape were investigated.

## Introduction

*Usnea* is one of the largest genera in the family *Parmeliaceae*, comprising about 350 species (Thell et al. 2012). They are characterized by having radially symmetric branches with a central chord consisting of an elastic cartilaginous strand of longitudinally orientated hyphae (Wirtz et al. 2006) and the presence of usnic acid in the cortex. The morphological variability among the species is a major challenge in the delimitation of the species (Clerc 1998). Motyka (1936) published a world monograph of *Usnea* in which the morphological features were the base for species recognition. However, morphological characters as used by modern taxonomists differ from those of Motyka (Clerc 1998). Revisions of the taxonomy of *Usnea* including descriptions of morphological characters and secondary chemistry as detected by thin layer chromatography have during the past 50 years been published from different parts of the world (Swinscow and Krog 1975; 1979, 1986, 1988; Clerc 1997; 1998, 2011; Ohmura 2001; 2012).

Molecular data have recently proved very useful in subgeneric and species recognition in *Usnea*, and the use of a combination of loci has resulted in good resolution (Truong et al. 2013).

Studies on *Usnea* species in Africa are few, but some species were described from Southern (Dodge 1956, 1957) and Eastern Africa (Swinscow and Krog 1975, 1979, 1986, 1988) based on morphology and chemistry data. Molecular work on African *Usnea* is limited, and only two sequences (ITS) have been published (Orock et al. 2012).

The genus *Eumitria* was described by Stirton (1882). It was characterized by having a tubular central axis ± filled with loose hyphae throughout the branches of the thallus. It was later considered a subgenus of *Usnea* (Motyka 1936; Ohmura 2001, 2002; Truong and Clerc 2013). Articus (2004), on the base of molecular data, resurrected it to generic level, which was also accepted by Divakar et al. (2017). Here, we treat *Eumitria* as a subgenus of *Usnea* since the latter forms a strongly supported monophyletic group that is morphologically well characterized (Ohmura and Kanda 2004; Wirtz et al. 2006; Thell et al. 2018).

*Eumitria* has been studied in Australia (Rogers and Stevens 1988; Stevens 1999), East Asia (Ohmura 2001, 2012), and South America and the Galapagos (Truong and Clerc 2013). The knowledge of *Eumitria* in Africa, however, is rather limited and mainly restricted to Central and East Africa (Motyka 1936; Dodge 1956; Swinscow and Krog 1974, 1986; Krog 1994). In these papers morphology and secondary chemistry (Table 1) were used in the descriptions.

**Table 1.** Table of *Usnea *subgenus *Eumitria *taxa from Africa (Dodge 1956; Swinscow and Krog 1974, 1986; Krog 1994) with their main chemistry indicated by X; protocetraric acid (PRO), fumarprotocetraic acid (FUM), diffractaic acid (DIF), salazinic acid (SAL), norstictic acid (NOR), constictic acid (CON), thamnolic acid (THA), psoromic acid (PSO), triterpenoids (TRI), virensic acid (VIR).

Species	Chemistry									
	PRO	FUM	DIF	SAL	NOR	CON	THA	PSO	VIR	TRI
*U. antiqua* Swinscow & Krog								X		
*U. baileyi* (Stirt.) Zahlbr.			X	X	X					
*U. baileyi var. pinnatifida* Swinsc. &Krog	X									
*U. baileyi var. planiuscula* Swinsc. &Krog	X									
*U. brunnescens* C.W. Dodge										
*U. cervicornis* C.W. Dodge	X	X	X							
*U. congdonii* Krog	X		X							X
*U. cristata* Mot.			X	X						
*U. elata* Mot.			X							
*U. firmula* (Stirt.) Mot.	X			X	X					
*U. liechtensteinii* Steiner	X									
*U. pulvinulata* C.W. Dodge	X		X	X	X					
*U. subcristata* C.W. Dodge			X	X		X				
*U. uluguruensis* Krog	X								X	
*U. welwitschiana* Mot.	X									
*U. zombensis* Krog							X			

Very little molecular work has been done on *Eumitria* worldwide, and even less on African material. Up to date (retrieved on the 24^th^ of January 2019) only one *Eumitria* ITS sequence (from *Usnea baileyi*), has been published from African material (Orock et al. 2012) and three ITS sequences are available from other parts of the world (Ohmura 2002). For *Usnea pectinata* Taylor so far only six verified sequences (three ITS, one mtSSU, one nuLSU and one MCM7) have been deposited in GenBank (Ohmura and Kanda 2004; Articus 2004; Truong et al. 2013), none of them from Africa. No other *Eumitria* species have so far been scrutinised by molecular work.

Mountain rainforests in Tanzania harbour a high diversity of *Usnea* species that remains understudied. This paper aims to present a phylogeny of *Eumitria* recently collected in Tanzania based on molecular information, supplemented by morphological and secondary chemistry data, providing up-to-date knowledge of this notoriously difficult and thus understudied group.

## Material and methods

### Taxon sampling

**Figure 1.**
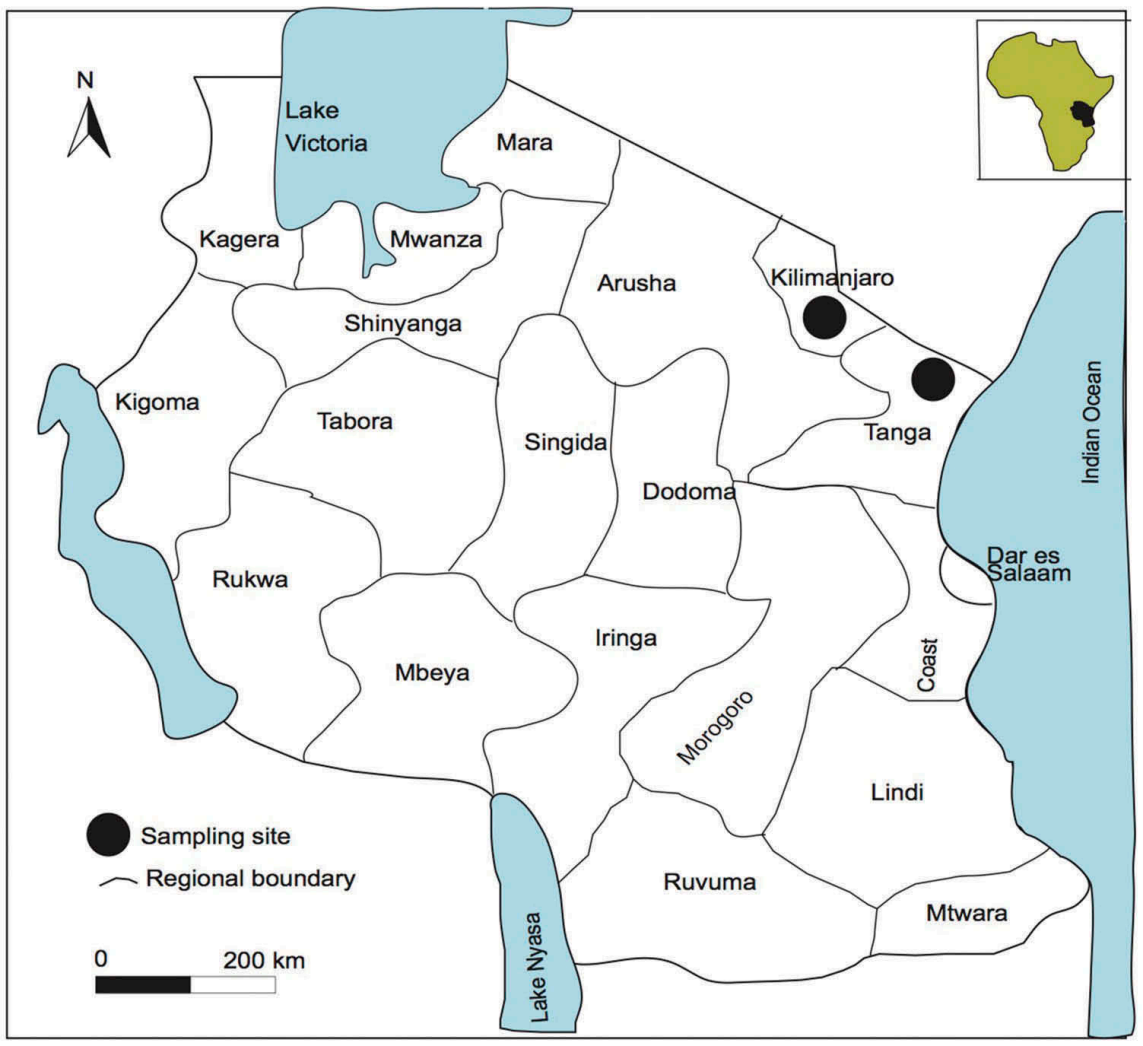
Map showing study sites in Tanzania indicated by dots.

This study is mainly based on materials collected by the first author in Korogwe (Tanga) and Kilimanjaro, Tanzania (Figure 1) in 2016 and 2017. Voucher specimens collected during field trips have been deposited in UPS with some duplicates in UDSM and G.

For the first dataset, i.e. the large dataset (LD), the ITS, nuLSU, MCM7 and RPB1 loci were included. This dataset comprised 49 specimens representing 15 species of *Usnea* (Table 2) selected for this study following the clades named by Truong et al. (2013). In addition, two species belonging to *Parmeliaceae* that served as outgroup (*Pleurosticta acetabulum* (Neck.) Elix & Lumbsch and *Lethariella cashmeriana* Krog were included.

**Table 2.** Species and GenBank accession numbers of sequences used in the DNA analyses. Newly produced sequences in bold.

		GenBank Accession numbers			
Species	Isolation No	ITS	nuLSU	MCM7	RPB1
*Lethariella cashmeriana*	Obermayer 8335	DQ980014	DQ923665		DQ923690
*Pleurosticta acetabulum*	MAF9914	AY581087	AY578953	JX136677	EF092147
*U. baileyi*	Y04488A	AB051050			
*U. baileyi*	Y4516	AB051051			
***U. baileyi***	**SGT 63**	MN080251			**MN080252**
***U. baileyi***	**SGT 65**	**MN080242**	**MN080261**	**MN097139**	
***U. baileyi***	**SGT 110**	**MN080250**	**MN080264**	MN097141	**MN098768**
***U. baileyi***	**SGT 112**	**MN080249**	**MN080265**		
***U. baileyi***	**SGT 118**	**MN080244**	**MN080266**		
***U. baileyi***	**SGT 119**	**MN080248**	**MN080267**		
***U. baileyi***	**SGT 120**	**MN080246**	**MN080262**	MN097142	**MN098771**
***U. baileyi***	**SGT 122**	**MN080247**	**MN080268**		
***U. baileyi***	**SGT 156**	**MN080245**	**MN080269**	MN097143	**MN098772**
***U. baileyi***	**SGT 157**	**MN080243**	**MN080263**		
*U. dasaea*	Y2842	AB051056			
*U. dasaea*	41	JQ837305	JQ837390	JQ837348	
*U. dasaea*	81	JQ837306	JQ837391	JQ837349	
*U. diffracta*	Y2889	AB051059			
*U. diffracta*	Y1124	AB051057			
*U. florida*	26	JN943538	JN939703		JN992584
*U. florida*	29	JN943535	JN939706		JN992581
*U. longissima*	Y2881	AB051643			
*U. longissima*	Y3844	AB051648			
*U. pectinata*	Y2989	AB051655			
*U. pectinata*	Y04373	AB051656	AB720729	AB720731	
***U. pectinata***	**SGT86**	**MN080233**	**MN080253**		**MN098765**
***U. pectinata***	**SGT87**	**MN080241**	**MN080254**		**MN098766**
***U. pectinata***	**SGT106**	**MN080234**			
***U. pectinata***	**SGT107**	**MN080240**	**MN080255**		
***U. pectinata***	**SGT109**	**MN080236**	**MN080256**	**MN097140**	**MN098767**
***U. pectinata***	**SGT114**	**MN080235**	**MN080257**		**MN098769**
***U. pectinata***	**SGT115**	**MN080237**	**MN080258**		
***U. pectinata***	**SGT116**	**MN080239**	**MN080259**		
***U. pectinata***	**SGT117**	**MN080238**			**MN098770**
*U. rubicunda*	Y3114	AB051659			
*U. rubicunda*	38	JQ837316	JQ837399	JQ837358	
*U. rubicunda*	39	JQ837317	JQ837400	JQ837359	
*U. subantarctica*	NW165	EF179805			EF179792
*U. subantarctica*	NW73	EF179806			EF179794
*U.sphacelata*	F964(NIPR)	AB103542			
*U.sphacelata*	F964(NIPR)	AB103543			
*U. subfloridana*	09–02124	JN943540	JN939701		N992586
*U. subfloridana*	09–02128	JN943537	JN939704		N992583
*U. trachycarpa*	F 1174029a	DQ235496	EF116570		EF179793
*U. trachycarpa*	411	DQ767964			EF193058
*U. trichodeoides*	Y.4413	AB051672			
*U. trichodeoides*	Y05316	AB720727	AB720728	AB720730	
*U. wamuthii*	28	JN943536	JN939705		JN992582
*U. wamuthii*	34	JN943530	JN939710		JN992576

The second dataset, the small dataset (SD), comprised the subgenus *Eumitria*, where all the above mentioned loci were combined to form 23 sets of sequences representing *Usnea baileyi* and *U. pectinata* (Table 2). Based on the results of LD analyses *Usnea longissima* Ach. and *U. trichodeoides* Vain. from the subgenus *Dolichousnea* Y. Ohmura were chosen as outgroup for the analyses of the SD.

#### Morphological, anatomical and chemical studies

The morphology of specimens was investigated using a stereomicroscope. For each specimen, three measurements were made: cortex, medulla, and central axis. These were made on longitudinal sections of branches at ×50 magnification. The relative thickness of cortex/medulla/axis of the total branch diameter (CMA) and the ratio of axis/medulla (A/M) of all the studied specimens were calculated according to Clerc (1984, 1987) and were ascribed to the categories defined by Clerc (2011). Percentages of the tubular part of the axis (TBA) were calculated according to Truong and Clerc (2013). In the species diagnostic features part, the CMA and TBA values are presented with their standard deviation. Observations of the anatomical structure of the cortex were made on thin hand-cut sections at ×400 as in Ohmura (2001).

Chemical analyses of all the studied specimens were performed by thin layer chromatography (TLC) in solvent A, B and C following Culberson and Ammann (1979), with solvent B modified according to Culberson and Johnson (1982). The chemical substances were considered as a “main substance” if they were present in all specimens studied of a given species.

### DNA extraction, PCR amplification and sequencing

Total DNA was extracted from freshly collected material less than three months old after having been kept at −20°C for a period of one month, using the DNeasy Plant Mini Kit (Quiagen, Hilden, Germany) following the manufacturer’s instructions. The material for extraction was selected carefully to avoid contamination. For each examined specimen, a branch piece about 1 cm long was used.

Total DNA was used for PCR amplifications. The primers used were ITS1F (Gardes and Bruns 1993), ITS4 (White et al. 1990); LROR and LR5 (Vilgalys and Hester 1990); MCM7-709 and MCM7-1349 (Schmitt et al. 2009); gRPB1-A and gRPB1-C (Matheny et al. 2002). The amplifications were carried out by using the AccuPower PCR PreMix (Bioneer, Daejeon, Korea), the reaction mixture consisting of 3 µl diluted DNA, 1.5 µl of each primer (10 mM), and water to a total volume of 20 µl. The thermal cycling parameters were: initial denaturation for 4 min at 95ºC, followed by 35 cycles of 1min at 94ºC, 1min at 54ºC, 45 s. at 72ºC, and a final elongation for 5 min at 72ºC. The PCR products were visualized by electrophoresis on 1.5% agarose gels. Products were purified using Illustra™ ExoStar buffer diluted 10×, following the manufacturer’s protocol. Sequencing was carried out by Macrogen (www.macrogen.com).

### Alignments and phylogenetic reconstructions

DNA sequences of ITS, nuLSU, MCM7 and RPB1 representing species in each of the subgenera of *Usnea* according to Ohmura and Kanda (2004) and Truong et al. (2013) were downloaded from GenBank, after assessment of their quality. The selected DNA sequences downloaded from GenBank, along with the newly produced sequences (Table 2), were assembled and edited using AliView (Larsson 2014) and aligned with MAFFT v.7 (https//mafft.cbrc.jp/alignment/server/).

Phylogenetic relationships and their posterior probabilities, for both the LD and the SD, were inferred/calculated using a Bayesian approach, and additional support values were estimated using Maximum Likelihood Bootstrap Support (MLbs). For the Bayesian analyses, the most likely models of evolution were estimated for each region separately using the Akaike Information Criterion (AIC) as implemented in Modeltest 3.7 (Posada and Crandall 1998).

For the LD the GTR + G model of evolution was employed for nuLSU, MCM7 and RPB1 whereas GTR + I + G was used for ITS. For the SD the GTR + G model was implemented for ITS, nuLSU and MCM7 while the K80 was employed in RPB1.

The Bayesian analysis was executed using MrBayes 3.2.6 (Ronquist et al. 2012), where two analyses of two parallel runs were carried out for 10 M generations. Each run included four chains, and trees were sampled every 1000 generations and 25% were discarded as burn in. All runs converged on the same average likelihood score and topology. Single gene analyses were performed to test the congruence among the four datasets (ITS, nuLSU, MCM7 and RPB1). A test was considered incongruent if a well-supported monophyletic group with posterior probability (PP) ≥ 0.95 was found to have low support (non- monophyletic) when different loci were used. No significant incongruence among the single gene trees (Supplementary figures S2A, S2B, S2C and S2D) was detected, hence the four matrices were concatenated. Further analyses were performed after concatenation using Sequence Matrix (Vaidya et al. 2011). Maximum likelihood estimates were carried out by RAxML version 8.2.10 using the GTR + G + I model of site substitution (Stamatakis 2014). The branch support was acquired by maximum likelihood bootstrapping (MLbs) of 1000 replicates (Hillis and Bull 1993), and MLbs ≥ 70% were considered to be significant.

The trees were visualized in FigTree version 1.3.1 (Rambaut and Drummond 2010).

## Results and discussion

### Phylogenetic studies

#### Infrageneric clades in Usnea

This study generated 62 new sequences of *Eumitria* (26 ITS, 20 nuLSU, 6 MCM7, 10 RPB1).

**Figure 2.**
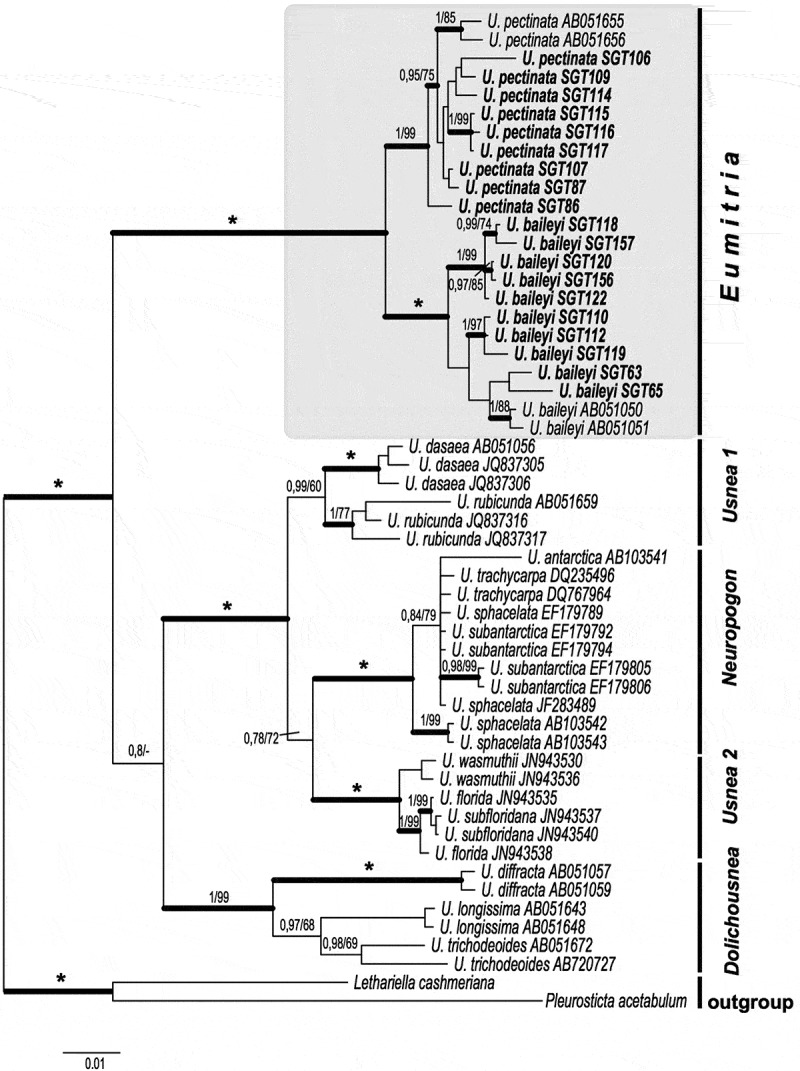
Consensus tree based on a Bayesian and ML analyses of concatenated ITS, nuLSU, RPB1 and MCM7 showing infrageneric clades in *Usnea*. The tree was rooted using two species *Pleurosticta acetabulum* and *Lethariella cashmeriana*. The two support values associated with each internal branch correspond to posterior probability (PP) and bootstrap support (bs) respectively. Branches in bold indicate a support of PP ≥ 95% and a MLbs ≥ 70%. An asterisk on a bold branch indicates that this node has a support of 100 % for both support estimates. A dash instead of a MLbs value indicates that the node of the Bayesian tree was not recovered by ML bootstrapping. Species groups (within annotation marks) are in accordance with Truong et al (2013). *Eumitria* is highlighted by a shaded box.

*Pleurosticta acetabulum* and *Lethariella cashmeriana* were chosen as outgroups. A consensus tree based on the LD (ITS, nuLSU, MCM7 and RPB1), with an indication of infrageneric clades of *Usnea*, is shown in Figure 2. *Usnea* contains well supported monophyletic clades agreeing with the circumscribed subgenera *Dolichousnea, Eumitria* and *Neuropogon*, in addition to *Usnea* 1 and *Usnea* 2, with *Eumitria* being the sister-clade to the other parts of *Usnea*. These results confirm those of Truong et al. (2013).

*Eumitria* in the SD is here represented by sequences of two species, *Usnea baileyi* and *U. pectinata*, with ten and nine samples respectively in the molecular study. nuLSU, MCM7 and RPB1 sequences are here for the first time reported for *U. baileyi. Usnea baileyi* and *U. pectinata* have previously been reported to belong in *Eumitria* based on molecular data (Ohmura and Kanda 2004; Articus 2004; Truong et al. 2013).

#### *Phylogeny of* Eumitria

**Figure 3.**
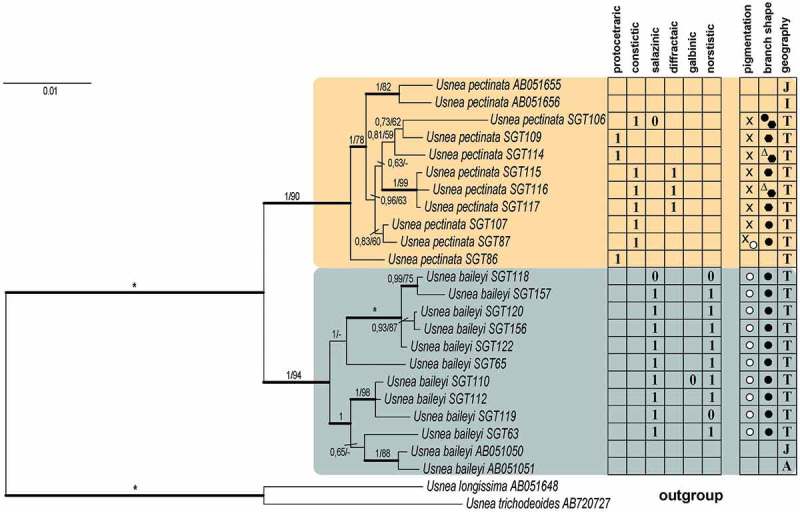
Consensus tree based on Bayesian and ML analyses of *Eumitria* species in Tanzania (ITS, nuLSU, RPB1 & MCM7). The two support values associated with each internal branch correspond to posterior probability (PP) and bootstrap support (bs) respectively. Branches in bold indicate a support of PP ≥ 95% and a MLbs ≥ 70%. An asterisk on a bold branch indicates that this node has a support of 100% for both support estimates. A dash instead of a MLbs value indicates that the node of the Bayesian tree was not recovered by ML bootstrapping. A: America, I: Indonesia, J: Japan, T: Tanzania 1: main chemical substance, 0 accessory chemical substance, x: dark brown pigmentation, big black dots: terete branch shape, triangles: alate branch shape, pentagon: ridged branch shape.

**Figure 4.**
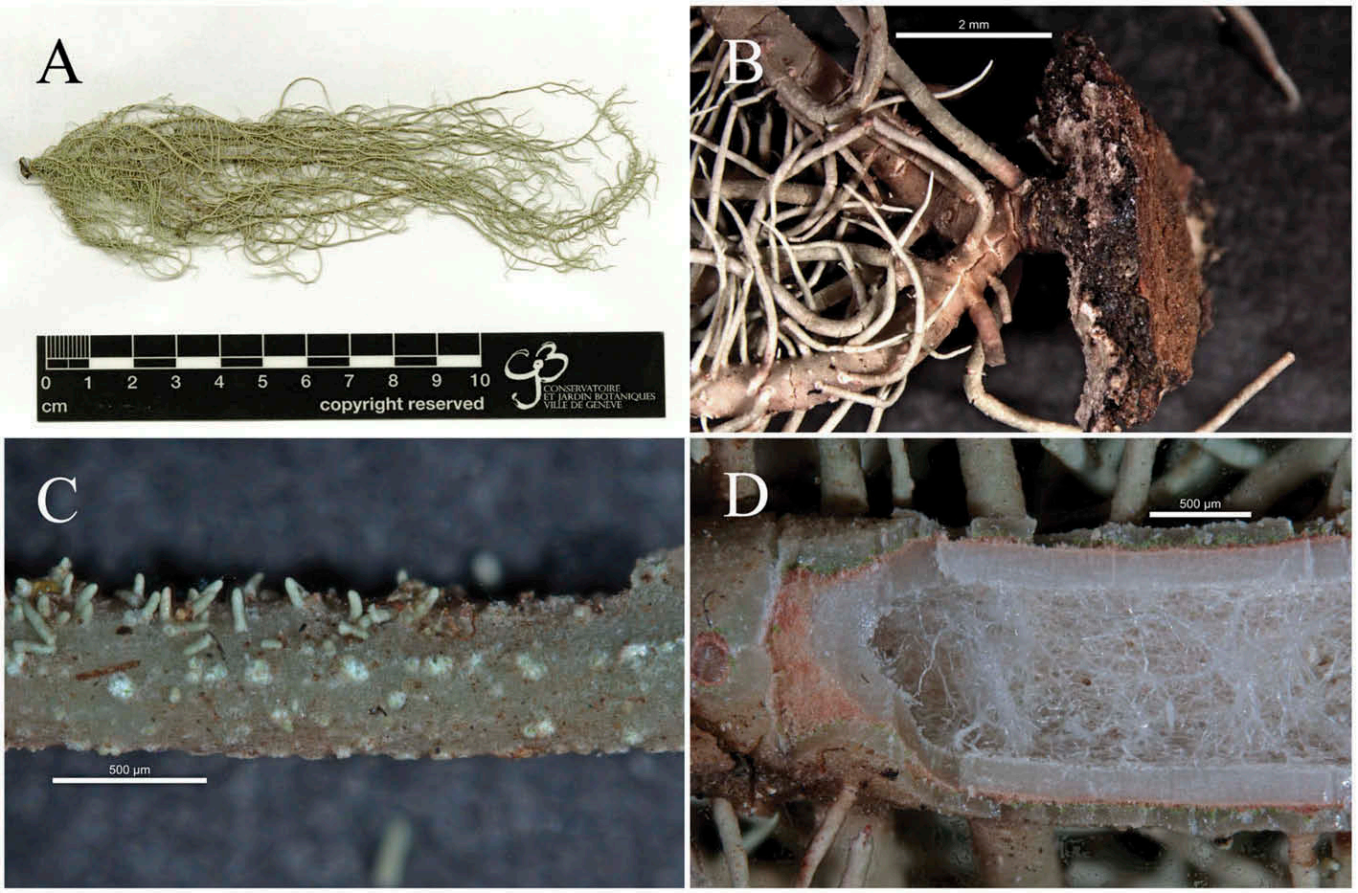
*Usnea baileyi*; (a): *Usnea baileyi *studied specimen (SGT 157), (b): blackish base, (c): soralia with short isidiomorphs (d): thin and shiny cortex, red subcortical pigment and tubular axis filled with loose hyphae.

**Figure 5.**
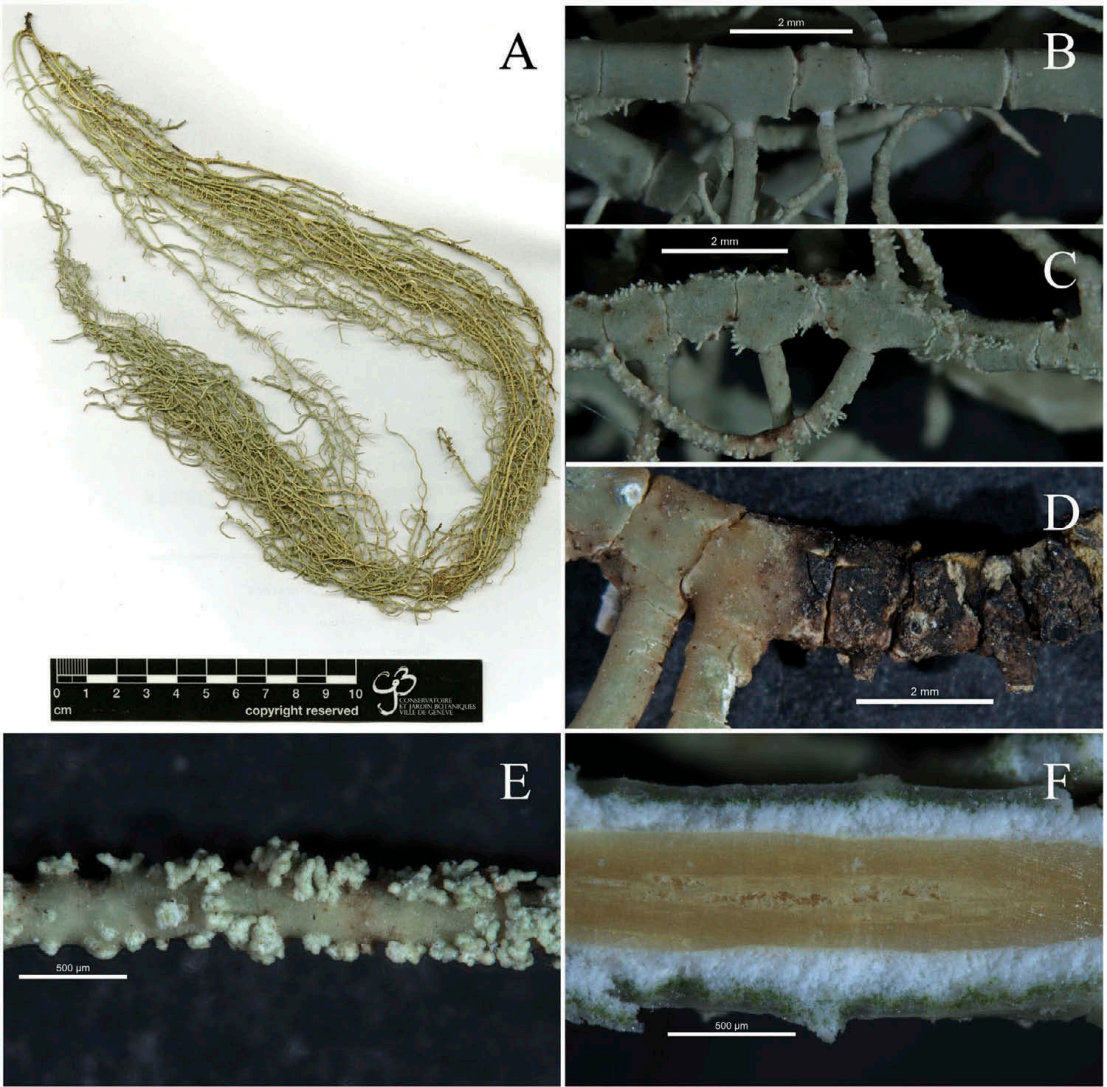
*Usnea pectinata*; (a): *Usnea pectinata* studied specimen (SGT 114), (b): smain branch cylindrical with terete segments, (c): main branch irregular with alate segments, (d): blackish base, (e): soralia with short isidiomorphs, (e): dark brown pigmented axis of main branch with some fistulose areas in the central part of the axis.

A phylogeny of *Eumitria* in Tanzania is presented in Figure 3 where *Usnea longissima* and *U. trichodeiodes*, were chosen as outgroup. The subgenus *Eumitria* is strongly supported in the molecular phylogeny (Figure 3). *Eumitria* was originally described as having a fistulose axis (Stirton 1882) and this feature was also emphasized by Motyka (1936). *Usnea baileyi* has a typical fistulose axis (Figure 4(d)). The close relationship between *Eumitria baileyi* and *U. pectinata* was, based on ITS, shown by Ohmura (2002), Articus (2004) and Wirtz et al. (2006). Truong and Clerc (2013), however stressed that the relationship of *Eumitria* needs more careful scrutiny. *Usnea pectinata* has a slightly fistulose axis on main branches (Figure 5(f)) and an overall morphology rather different from that of *U. baileyi*, but Ohmura (2002) considered it to belong to *Eumitria* as molecular data suggested a strong relationship with *U. baileyi*. The molecular data presented here as based on four loci strongly support the inclusion of *Usnea pectinata* in *Eumitria* (Figures 2 and 3).

RPB1 (Supplementary material (SM): Figure S2D) contributed to a better phylogenetic resolution than ITS (SM: Figure S2A) and MCM7 (SM: Figure S2C). The reliability of RPB1 as a phylogenetic marker has been emphasized in studies of *Parmeliaceae* such as in parmelioid and cetrarioid lichens (Crespo et al. 2010; Nelsen et al. 2011). The sequences of *U. pectinata* produced in this study form a strongly supported monophyletic clade (PP 1, MLbs 90) with the sequences *U. pectinata* available from GenBank (Figure 3). The strong relationship between *Usnea baileyi* and *U. pectinata* is also demonstrated. This is a further step in treating *Eumitria* species from Tanzania and Africa, and for the first time comprehensive molecular data is supplied.

### The species

*Usnea baileyi* (Stirt.) Zahlbr. (Figure 4)

#### Diagnostic notes

Detailed descriptions of this species were given by Ohmura (2001) and Truong and Clerc (2013). *Usnea baileyi* has a sorediate, shrubby to sub-pendulous thallus with cylindrical branches often tapering towards the apices, and with few annulations (Figure 4(a)). The base is concolorous or blackish with cracks below the first ramification (Figure 4(b)). Numerous soralia with short isidiomorphs cover the branches (Figure 4(c)). The cortex is shiny in section and thin to moderately thin (5.7–7.8%). A red subcortical pigment was observed (Figure 4(d)). The medulla is thin ranging from 3.6 to 5.7%. The axis is thick (78–82%), most of it occupied by a tubular section (43–46%) filled with loose hyphae (Figure 4(d)). These values differ from those reported by Ohmura (2001) and Truong and Clerc (2013) as shown in Table 3.

**Table 3.** Comparison of *U.* *baileyi* anatomical characters. Mean (italic), standard deviation and extreme values (in parenthesis) are shown.

Anatomical characters	Japan (Ohmura 2001)	South America and the Galapagos (Truong and Clerc 2013)	Tanzania
% C	(3.3) – 4.9–*6.6*–8.3 – (9.2)	3.0–5.5	(4.4) – 5.7–*7.0*–7.8 – (8.2)
% M	(3.0) – 3.2–*5.3*–7.4 – (12)	1.5–3.0	(2.8) – 3.6–*4.2*–5.7 – (6.2)
% A	(58) – 70–*76* – 82 – (86)	83.0–91.5	(70) – 78–*81.79*–82 – (88)
% TBA	–	67–83	(38) – 43–*53.33*–46 – (84)

#### Chemistry

In addition to usnic acid, all the specimens examined contained salazinic and norstistic acids as main substances. These substances have previously been reported to occur in *Usnea baileyi* from East Africa (Swinscow and Krog 1974). A trace of galbinic acid was detected in one specimen (SGT 110), this is for the first time reported for *U. baileyi*.

#### Distribution and ecology

*Usnea baileyi* has a subtropical-tropical distribution and a wide ecological range (Truong and Clerc 2013). The specimens studied were found on twigs of *Isoberlinia scheffleri* and *Alablankia stomanii* twigs in an altitude of 1227–1307 m in Korogwe (Tanga), and also on *Makaranga kilimanjarika* twigs at 1954 m in Marangu (Kilimanjaro).

*Specimens examined*: Tanzania, **Tanga**, about 16.4 km from Korogwe district in the Usambara Mountains, 5°04’15 “S 38°24’02“E, 1227 m (SGT 63, SGT 65), 5°04’19“S 38°24’16”E, 1307 m (SGT 110, SGT 112, SGT 118, SGT 119, SGT 120, SGT 122), 2017. Tanzania, **Kilimanjaro**, Marangu route in the upper montane *Podocarpus* forests, 5°02’31”S 37°30’56”E, 1954 m (SGT 156, SGT 157), 2017.

*Usnea pectinata* Taylor (Figure 5)

#### Diagnostic notes

For a detailed description of this species see Ohmura (2001).

*Usnea pectinata* has a pendulous sorediate thallus (Figure 5(a)); branches have annulations and vary from smooth and cylindrical to irregular/ridged and more or less alate (Figure 5(b and c)). The base is blackish (Figure 5(d)) or not. The branches are covered by numerous soralia with isidiomorphs (Figure 5(e)). The cortex is shiny in section and moderately thin to moderately thick (6.6–8.8%). The medulla is thin ranging from 11.8–13.8%. The axis is moderately thin to moderately thick (44–46.3%), solid, varying in colour from white to pale to deep brown (Figure 5(f)).

#### Chemistry

Usnic acid was found in all specimens, but three chemotypes were observed as detected by TLC in the eight studied specimens. Only main substances were considered. Two specimens contained constictic acid; three contained constictic and diffractaic acid while the other three specimens contained protocetraric acid only. Ohmura (2001) reported norstictic, menegazziaic, stictic and constictic acids for *U. pectinata* specimens in Japan. Further investigations of the chemotypes in *U. pectinata* with additional material are in progress.

#### Distribution and ecology

Tropical (Ohmura 2001). In Tanzania found on twigs of *Drypetes usambarica, Alablankia stomanii* and *Ficus soningiae* at an altitude between 1227–1307 m.

*Specimens examined*: Tanzania, **Tanga**, about 16.4 km from Korogwe district in the Usambara Mountains, 5°04’15“S 38°24’02“E, 1227 m (SGT 86, SGT 87), 5°04’15”S 38°24’02”E, 1307 m (SGT 106, SGT 107, SGT 109, SGT 114, SGT 115, SGT 116, SGT117), 2017.

## Conclusion

Phylogenies of *Eumitria* species from Tanzania based on concatenated data sets were presented using Bayesian and maximum likelihood analyses (Figures 2 and 3). A total of 62 new sequences of *Eumitria* (26 ITS, 20 LSU, 6 MCM7, 10 RPB1) were generated, with nuLSU, MCM7 and RPB1 reported for the first time for *Usnea baileyi*. Variation in secondary chemistry and anatomical characters for *U. pectinata* was documented. For the first time morphology, chemistry, and molecular data were used in the analyses of *Eumitria*.
