# Supplementary material for: Phylogeny of the subgenus Eumitria in Tanzania
Source: Mycology. 2019 Jun 30;10(4):250–60. doi: 10.1080/21501203.2019.1635217 (PMC6781463; doi:10.1080/21501203.2019.1635217)
Supplement: Supplemental Material [file TMYC_A_1635217_SM8698.zip › Supplimentary_captions.docx]

SM (S2A): Tree based on a Bayesian analysis of ITS. The support values associated with each internal branch correspond to posterior probability (PP).

SM (S2B): Tree based on a ML analysis of ITS. The support values associated with each internal branch correspond to bootstrap support (bs).

SM (S2C): Tree based on a Bayesian analysis of nuLSU. The support values associated with each internal branch correspond to posterior probability (PP).

SM (S2D): Tree based on a ML analysis of nuLSU. The support values associated with each internal branch correspond to bootstrap support (bs).

SM (S2E): Tree based on a Bayesian analysis of MCM7. The support values associated with each internal branch correspond to posterior probability (PP).

SM (S2F): Tree based on a ML analysis of MCM7. The support values associated with each internal branch correspond to bootstrap support (bs).

SM (S2G): Tree based on a Bayesian analysis of RPB1. The support values associated with each internal branch correspond to posterior probability (PP).

SM (S2H): Tree based on a ML analysis of RPB1. The support values associated with each internal branch correspond to bootstrap support (bs).
