# Supplementary figures and images for: Phylogeny of the subgenus Eumitria in Tanzania
Source: Mycology. 2019 Jun 30;10(4):250–60. doi: 10.1080/21501203.2019.1635217 (PMC6781463; doi:10.1080/21501203.2019.1635217)

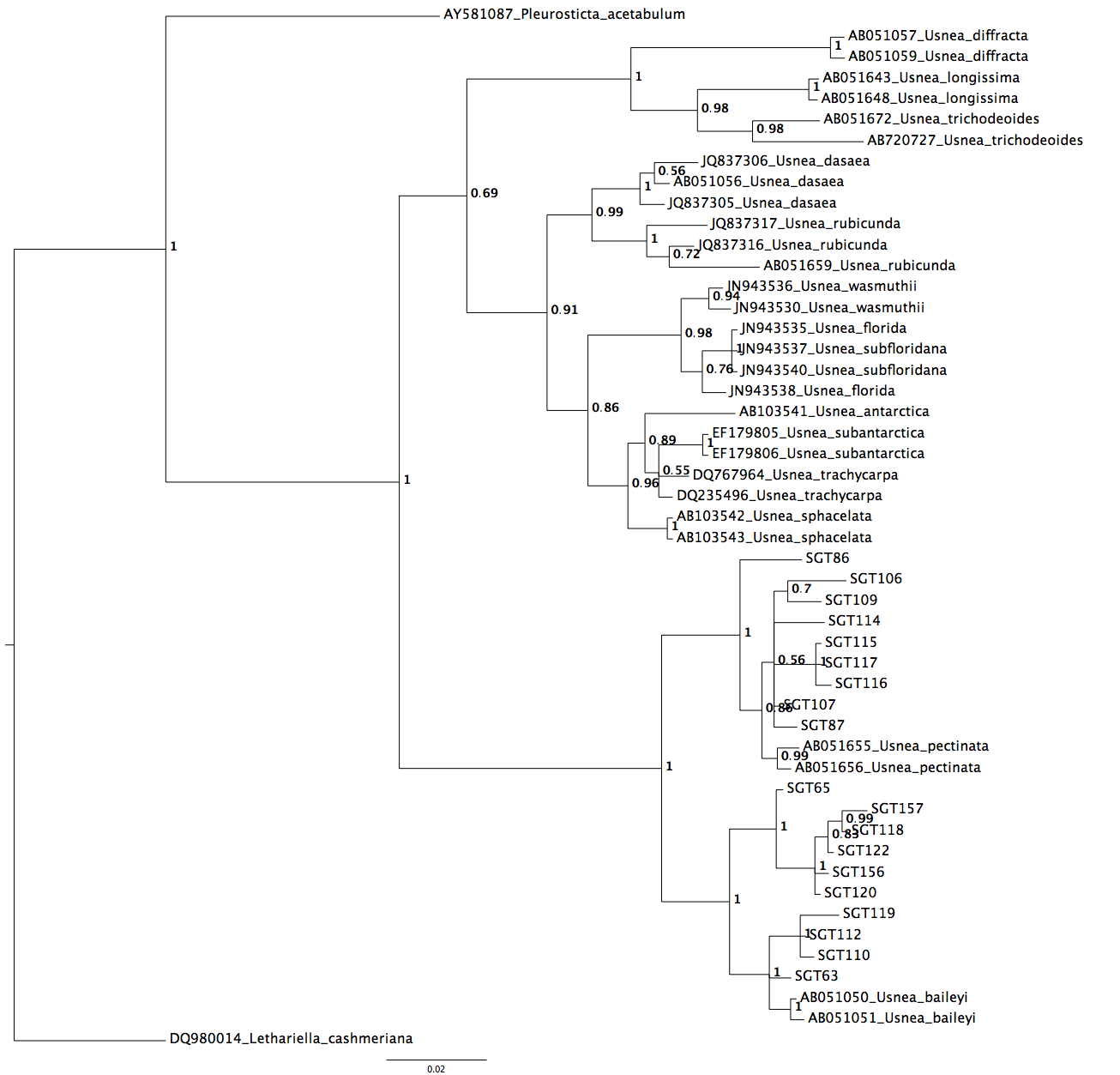

Supplement: Supplemental Material [file TMYC_A_1635217_SM8698.zip › S2A.png]

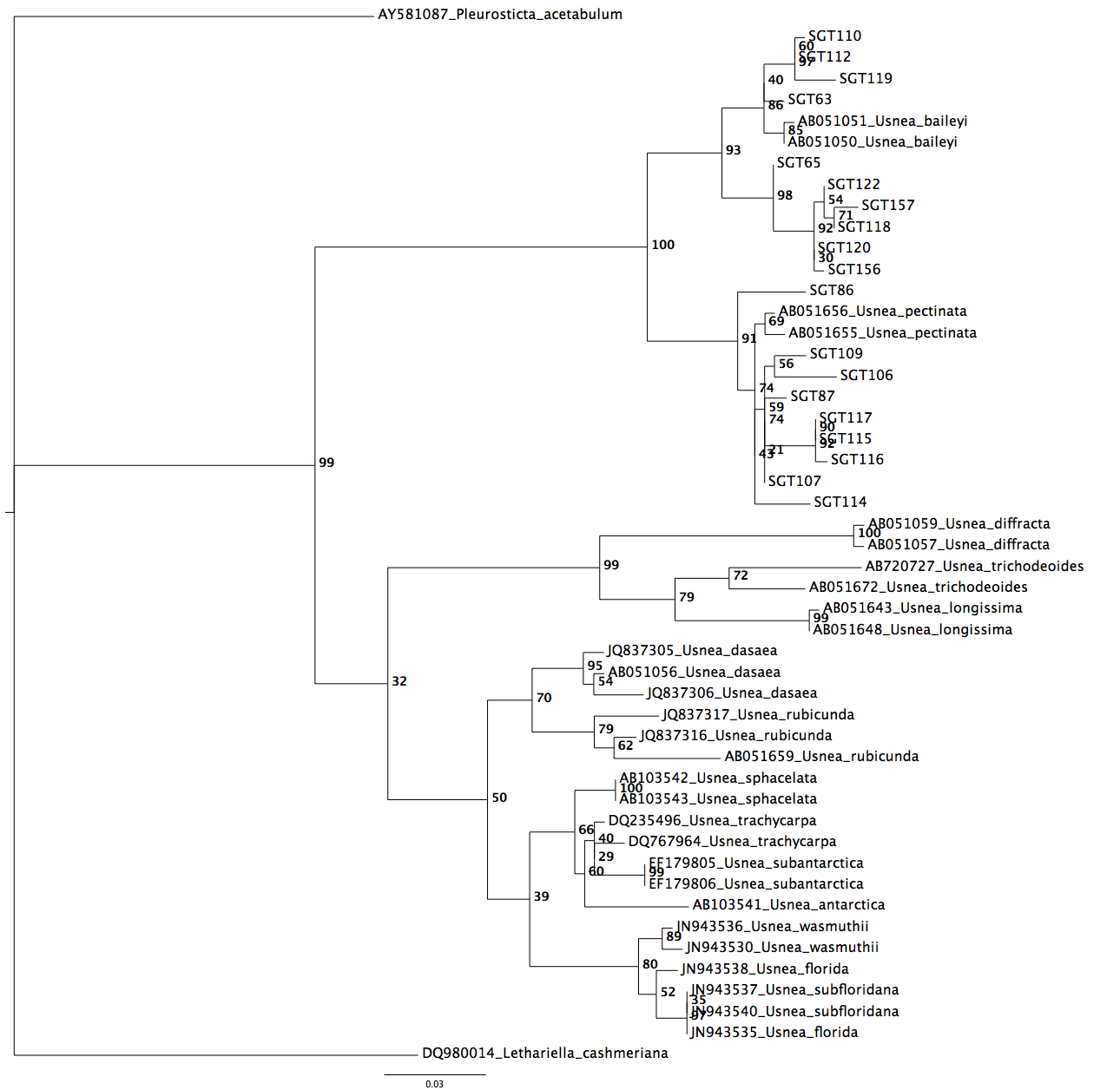

Supplement: Supplemental Material [file TMYC_A_1635217_SM8698.zip › S2B.png]

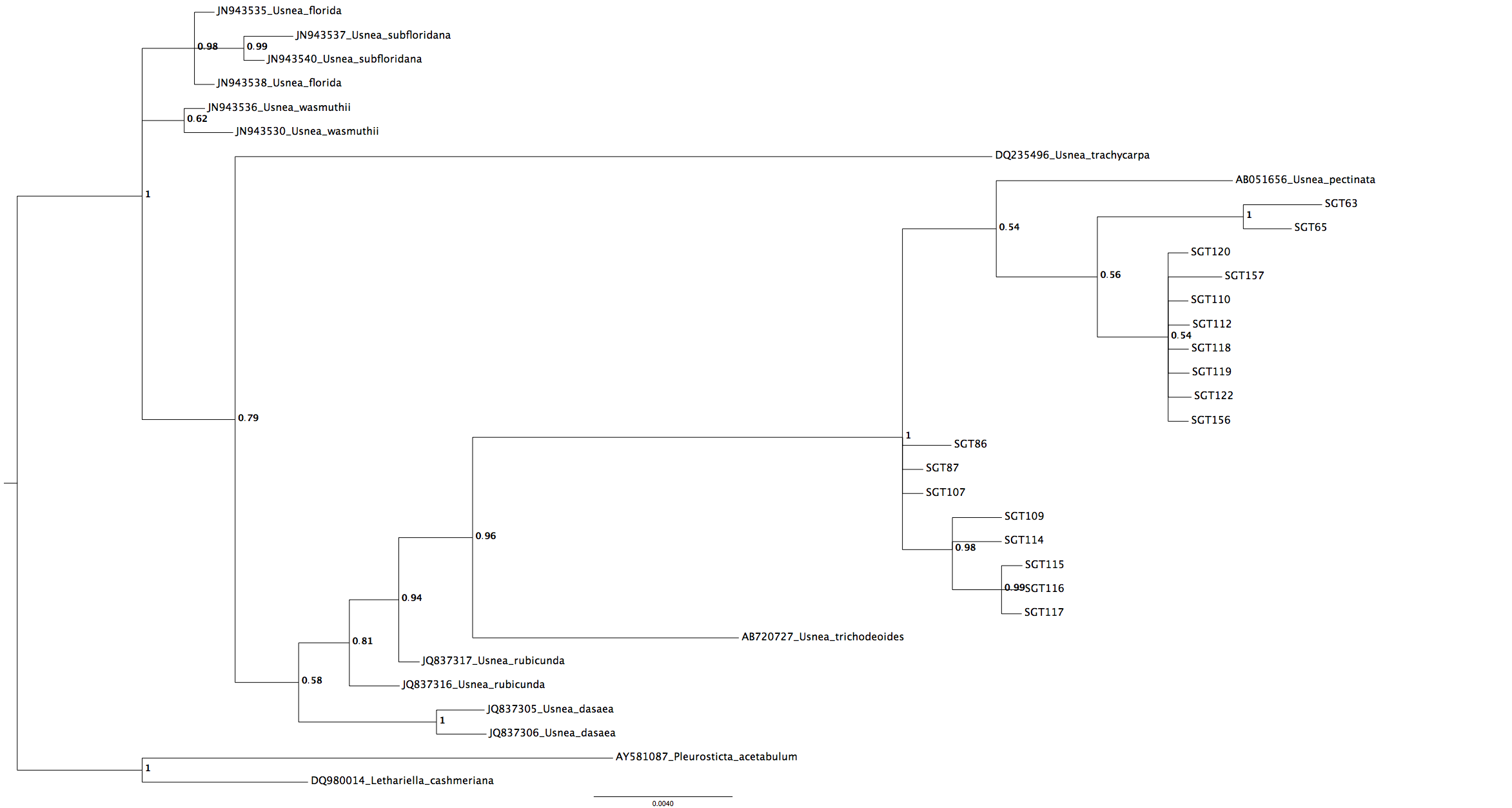

Supplement: Supplemental Material [file TMYC_A_1635217_SM8698.zip › S2C.png]

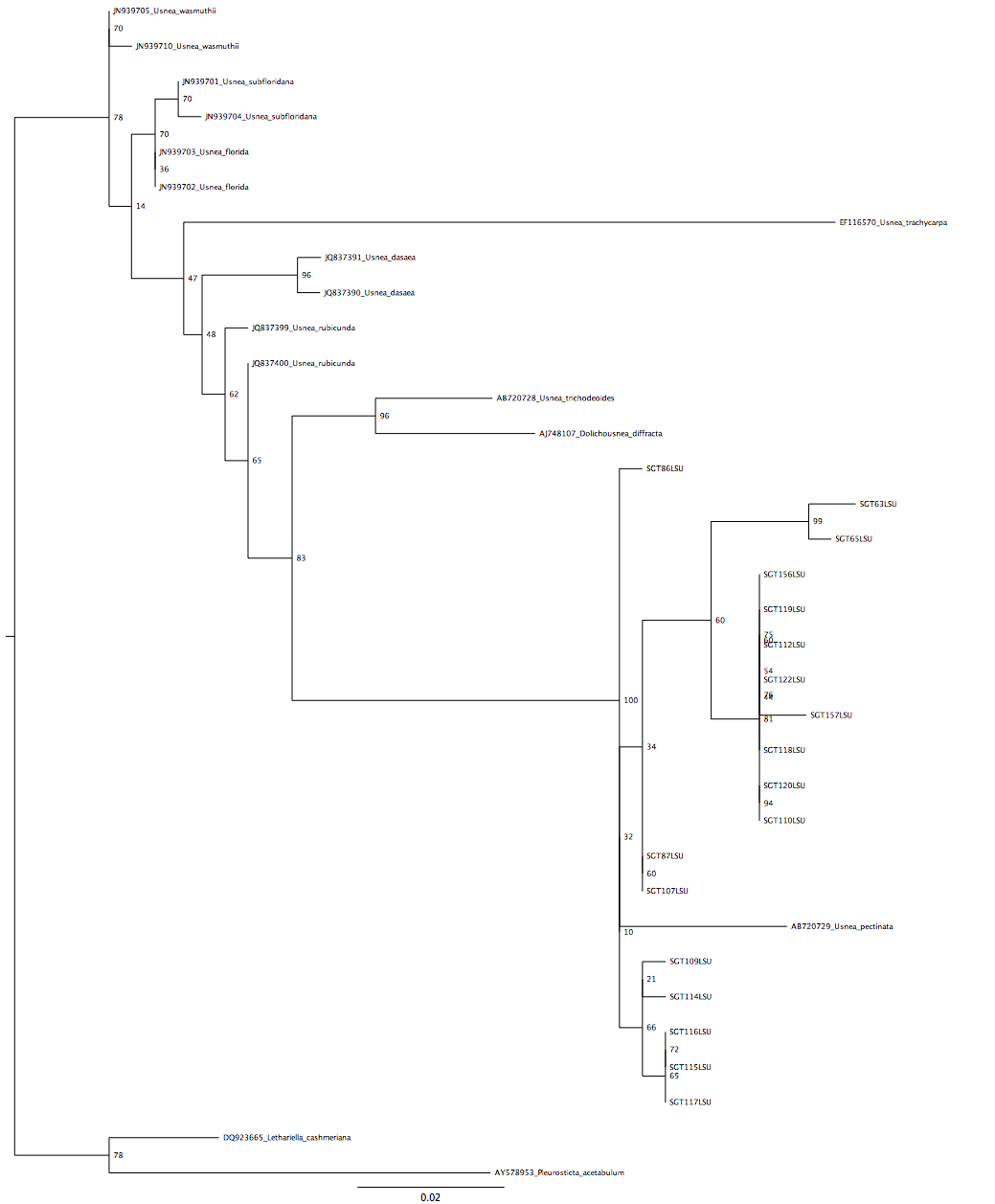

Supplement: Supplemental Material [file TMYC_A_1635217_SM8698.zip › S2D.png]

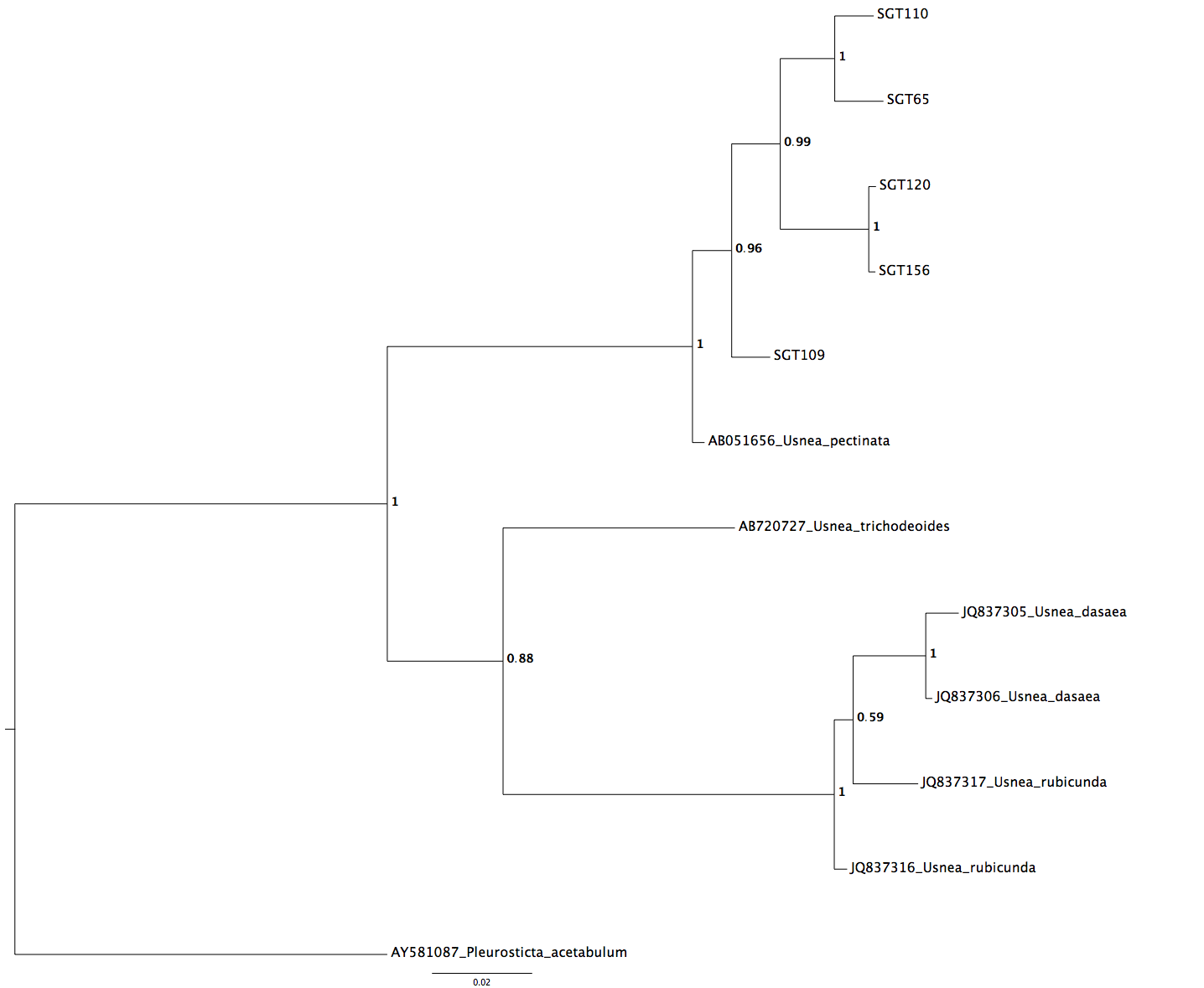

Supplement: Supplemental Material [file TMYC_A_1635217_SM8698.zip › S2E.png]

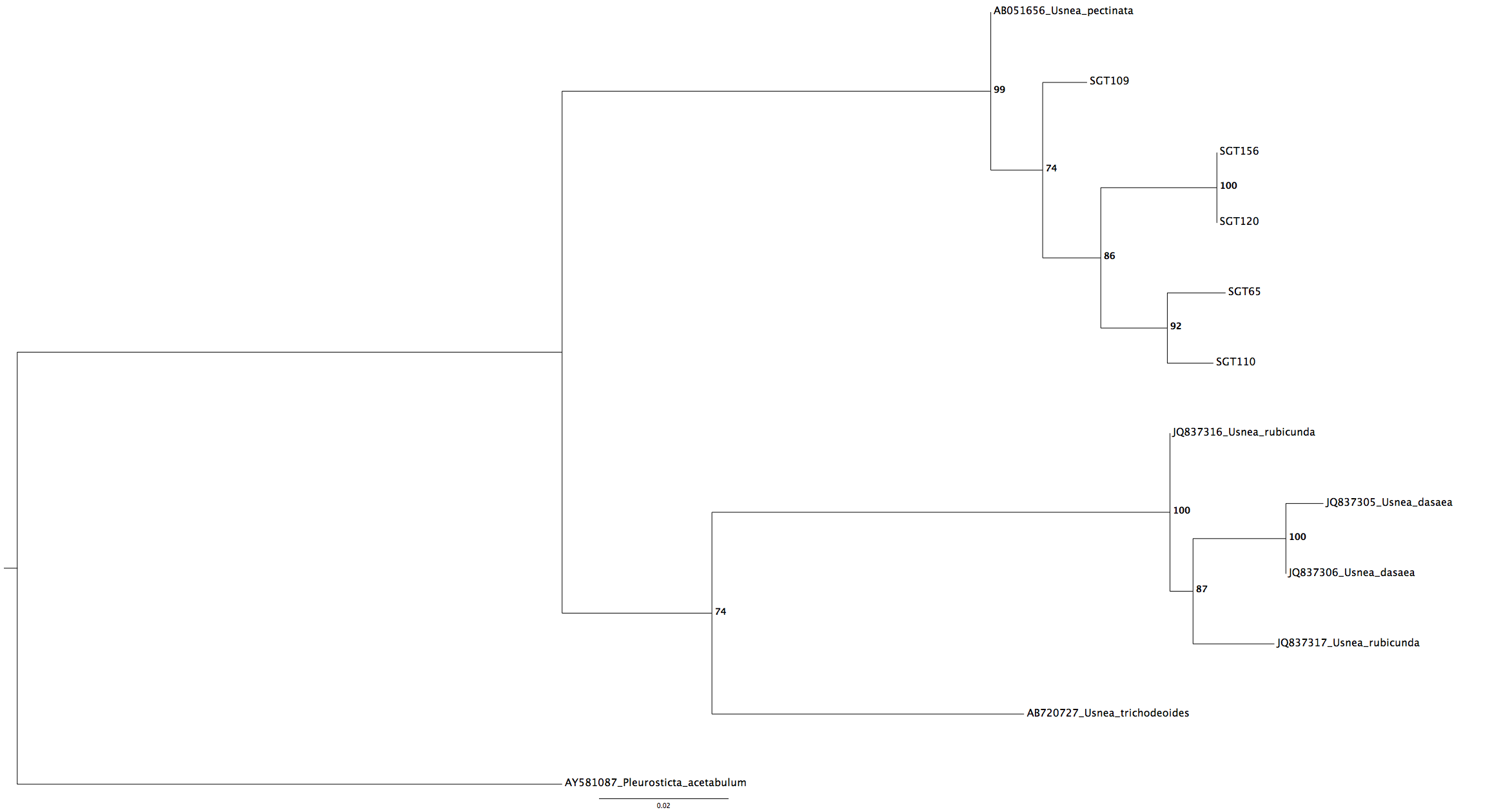

Supplement: Supplemental Material [file TMYC_A_1635217_SM8698.zip › S2F.png]

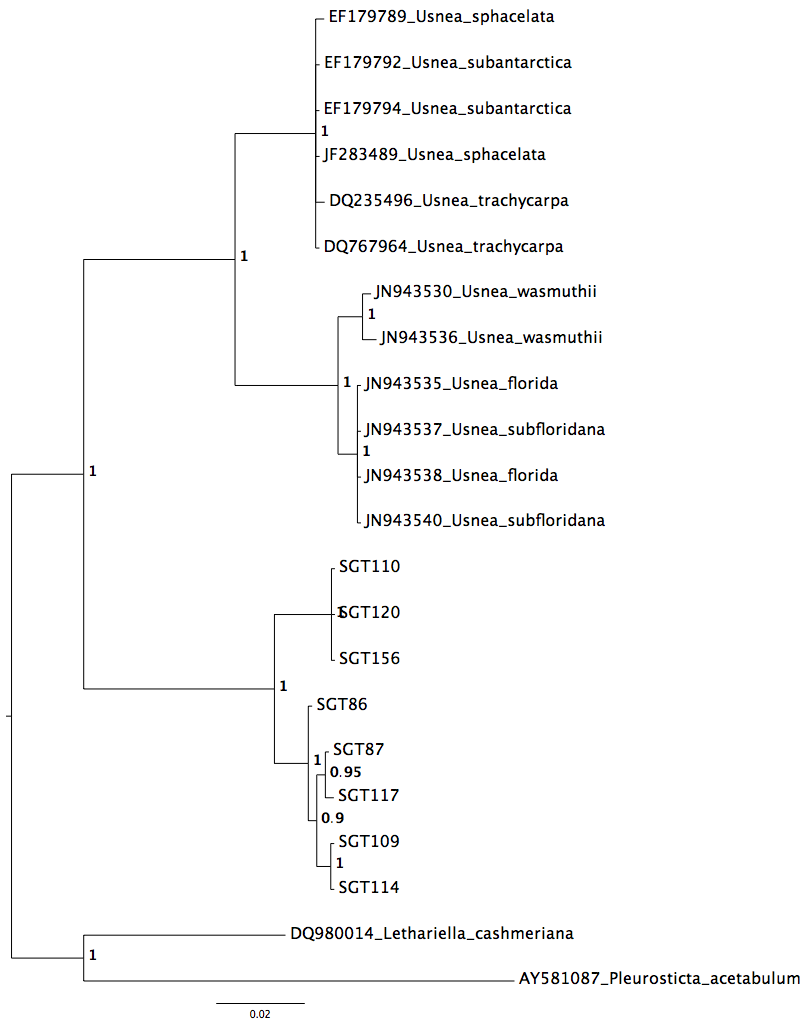

Supplement: Supplemental Material [file TMYC_A_1635217_SM8698.zip › S2G.png]

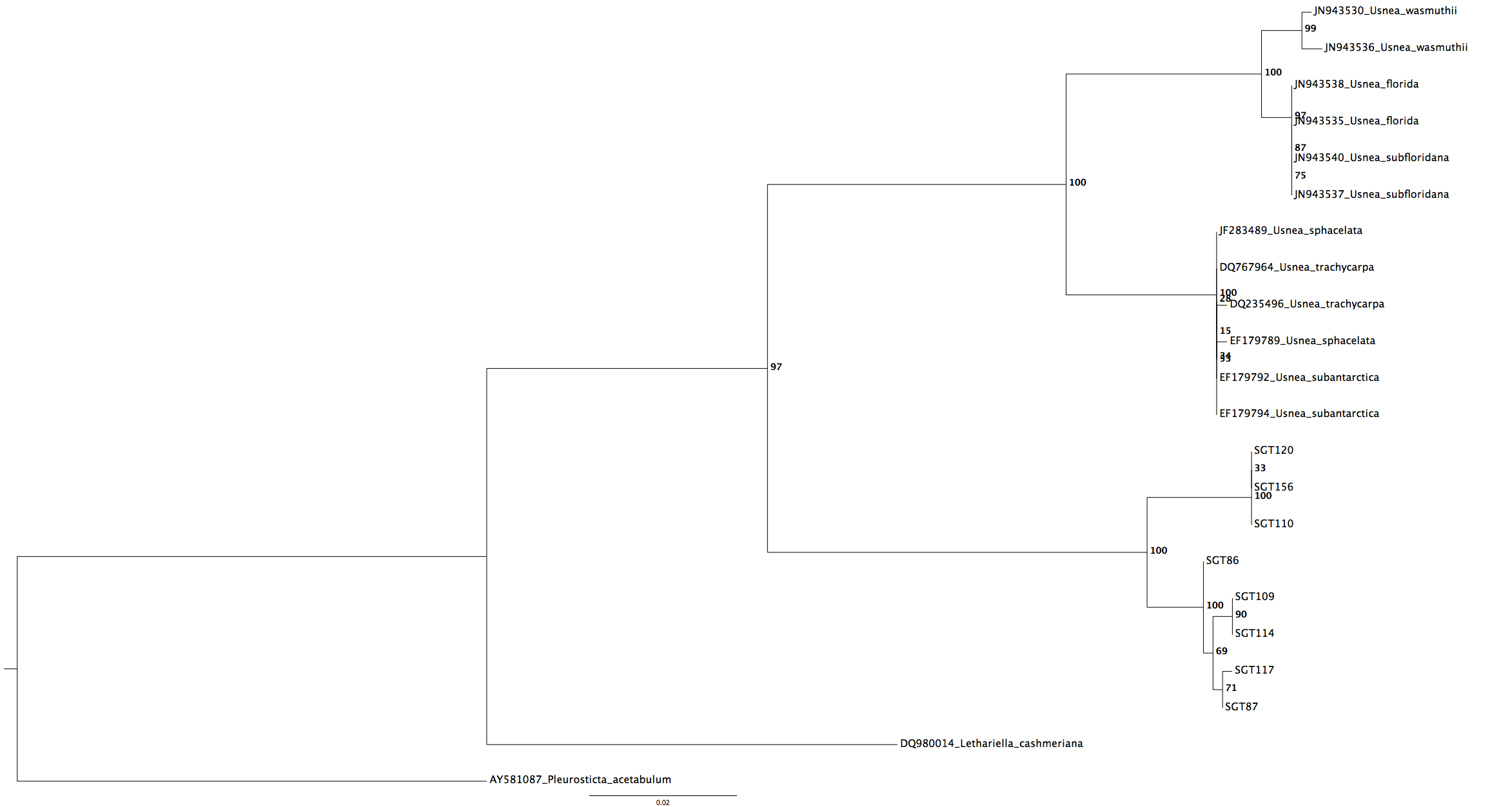

Supplement: Supplemental Material [file TMYC_A_1635217_SM8698.zip › S2H.png]
